# The Best Cut-Off Value for HbA1c as a Screening Tool in Iranian Women With Gestational Diabetes Mellitus

**Published:** 2017-03

**Authors:** Seyedeh Neda Mousavi, Koorosh Kamali, Motahareh Mirbazel, Maryam Jameshorani

**Affiliations:** 1 Department of Biochemistry and Nutrition, School of Medicine, Zanjan University of Medical Sciences, Zanjan, Iran; Metabolic Diseases Research Center, Vali-e-Asr Hospital, Zanjan University of Medical Sciences, Zanjan, Iran; 2 Department of Public Health, School of Public Health, Zanjan University of Medical Sciences, Zanjan, Iran; 3 Metabolic Diseases Research Center, Vali-e-Asr Hospital, Zanjan University of Medical Sciences, Zanjan, Iran

**Keywords:** Glucose Tolerance Test, Gestational Diabetes Mellitus, Glycosylated Hemoglobin A

## Abstract

**Objective:** Gestational diabetes mellitus (GDM) is a prevalent disorder which increases maternal and fetal complications. The oral glucose tolerance test (OGTT) is a traditional, time -consuming and intensive test which is poorly tolerated by pregnant women. To date, increasing evidence considered HbA1c as a screening tool and reported various cut-off values in different populations. In alignment with existing literature, we determined for the first time, the optimal cut-off value for HbA1c in Iranian women with GDM.

**Materials and methods:** This case-control study was conducted in Valie-Asr hospital between June 2015 and March 2016. A total of 200 pregnant women who were diagnosed with GDM were selected as study cases. For the control group, 200 healthy women were randomly selected. Fasting blood samples were taken for biochemical analysis, and OGTT was done in all participants. Demographic and anthropometric indexes were measured. Performance of the HbA1c test was analyzed by the Receiver Operating Characteristic (ROC) curve, and the sensitivity and specificity for different HbA1c cut-off points were calculated subsequently.

**Results:** Analysis showed that the mean age (p < 0.001) and BMI (p < 0.001) were significantly higher in the GDM group compared to those in non-GDM pregnant women. GDM participants reported positive family- and previous history of GDM more than healthy pregnant women (p = 0.04 and p < 0.001, respectively). All the markers for Lipid profile were significantly different between the two groups (p = <0.001) except for total cholesterol. The rate of Caesarean section and neonate’s Apgar score were not significantly different between the two groups. The best equilibrium between sensitivity (80%) and specificity (76%) for HbA1c was 5.05%.

**Conclusion:** Our results suggest that pregnant women with HbA1c of ≥ 5.05% should proceed with an OGTT. Further investigations with larger sample size are needed to provide more robust evidence for the diagnostic and screening value of HbA1c in identifying pregnant women with GDM.

## Introduction

Gestational diabetes mellitus (GDM) is a prevalent disorder, resulting in more than 200,000 cases annually (1). It increases the risk of maternal problems, such as gestational hypertension, preeclampsia and cesarean section rate (2, 3). GDM can also increases fetal complications including hyperinsulinemia, macrosomia (often defined as a baby weighing more than 4000 grams), and consequently delivery complications, such as shoulder dystocia. The neonate is more likely to require admission to NICU or to experience respiratory distress syndrome, and metabolic complications, including hyper-bilirubinemia and hypoglycemia (4). The severity of adverse outcome depends on the time of GDM diagnosis and glycemic control. In fact, a linear correlation has been seen between maternal blood glucose level and various neonatal complications (5). Despite the well-established complications related to GDM, there is still substantial argument about its diagnosis (6, 7). According to the current revised version of guidelines published in 2013, an increase in the GDM incidence was detected (5). Traditional guidelines recommend that the OGTT (oral glucose tolerant test) containing 75g oral glucose at 24-28 weeks of gestation is a test for GDM diagnosis. It is a time-consuming and invasive method which is done for fasting pregnant women. In addition to these difficulties, there is a considerable inconsistency in the OGTT cut-offs for the GDM diagnosis (3, 8). The American Diabetic Association (ADA) and World Health Organization proposed that HbA1c test should be used for the diagnoses of type 2 diabetes, but not for GDM (9, 10). Over the erythrocyte life span, plasma glucose irreversibly is bound to hemoglobin (Hb) and determines the HbA1c level. It is a non-fasting blood test and reflects glucose levels over the last 3 month. It is shown that compared to glucose test, HbA1c has more reliability with less than 6% inter-laboratory variation. HbA1c has also better standardization between assays, as well as less pre-analytical and intra-individual variations (11, 12). 

Due to the difficulties in OGTT method, as well as various cut-offs for HbA1c in different populations (because of difference in races and screening criteria) (13-17), newer acceptable and accessible tests are needed in each population. The aim of the present study is to assess the optimal cut-off value of HbA1c, in order to be used as a potential substitute for OGTT, in GDM screening for Iranian population.

## Materials and methods

The present case-control study was approved by the ethics committee of Metabolic Research Center at Zanjan University of Medical Sciences. It was performed in accordance with ethical standards, laid down in the 1964 Declaration of Helsinki, and its later amendments or comparable ethical standards. The study was carried between June 2015 and March 2016. Pregnant women, without previous history of GDM or DM, attending the Valie-Asr Hospital to perform OGTT at the 24-28 week of pregnancy, were invited to participate in this study. Totally 200 GDM pregnant women who were diagnosed according to ADA/WHO 2013 criteria (fasting, 1 h and 2 h blood glucose more than 92 mg/dl, 180 mg/dl and 153 mg/dl) were selected as study cases, and 200 non GDM women were recruited for the control group (9, 10). All subjects provided their signed consent before the study enrollment. Demographic data including age of participants, gestational age, obstetric history, smoking, family history of chronic diseases such as type 2 diabetes, cardiovascular disease, hypertension, and history of high risk pregnancies were recorded. Patients with abnormal thyroid hormone concentration, anemia, presence of hemoglobinopathy, intake of supplements and/or medications that could affect body weight and/or energy expenditure, having a prescribed diet for weight control, smoking, diagnosis of chronic diseases including inflammatory diseases, heart, chronic liver and renal failure, cancer, acute myocardial infarction, diabetes, stroke, or serious injuries, and any other conditions that were not suitable for the trial as evaluated by the physician, were excluded from the study. Body weight and height were taken by using a calibrated Seca scale (to the nearest 100 grams) and a wall-mounted Seca scale (to the nearest 0.5 cm). BMI (Body Mass Index) was calculated according to the formula: BMI = weight/ height^2^ (kg/m^2^). Blood samples for all subjects were taken from the antecubital vein after 10-12 hour of fasting, and between 0700 and 0800 AM for biochemical measurements. Fasting, 1hour and 2 hour blood glucose were measured by an enzymatic method (Pars Azmoon Co. kit, Tehran, Iran) using Liasys autoanalyzer. Serum levels of HbA1c were measured by HPLC method (KNAUER, Germany, ser.No: 211133). 

***Statistical analysis: ***All data were expressed by means ± SD. P < 0.05 was considered significant. IBM SPSS Statistics software (version 18; IBM Corp) was used for data analysis. Normal distribution of the variables was checked by Kolmogorov Smirnov Test**. **Normal distributed data were compared by the independent sample t-test, whereas non-normal distributed data were compared by the Mann-Whitney Wilcoxon test. Person test was used to measure correlations between the variables. Chi-square test was used for qualitative data analysis. Performance of the HbA1c test in GDM screening (considering the OGTT as the reference test) was analyzed by the Receiver Operating Characteristic (ROC) curve. A ROC statistics based upon logistic regression analysis considering the covariates “previous history of GDM”, “age” and “BMI” in the model were also carried out. Sensitivity and specificity for different HbA1c cut-off points were calculated.

## Results

**Figure 1:**
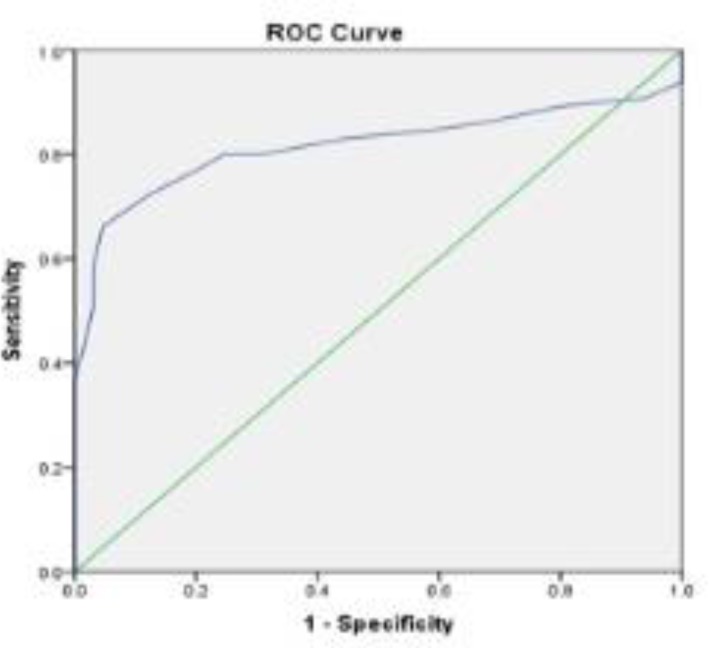
ROC curve (95% CI = 0.734-0.898; p < 0.001)

As shown in table 1, the age of GDM patients was significantly higher than controls (p < 0.001). Positive family, as well as previous GDM history were significantly higher in the GDM pregnant mothers than the control group (p = 0.04 and p < 0.001, respectively). More pregnant women in the GDM group underwent the caesarean section than controls (p = 0.02). Mean of BMI in the GDM group was significantly higher compared with the non-GDM group (p < 0.001). More participants in the GDM group had history of GDM in previous pregnancies, compared with the controls (p < 0.001). TG (p < 0.001), LDL.C (p < 0.001) and HDL.C (p = 0.001), but not TC were significantly different between the two groups. Significant correlation was shown between HbA1c and neonatal Apgar, as well as HbA1c and neonatal height (p = 0.03 and p = 0.04, respectively). A ROC analysis (Figure 1, Table 2) found an AUC of 0.82 (95% CI = 0.734-0.898, p < 0.001) and the best equilibrium between sensitivity and specificity for HbA1c was 5.05.

**Table 1:** GDM patient’s characteristics compared with controls

**Variables **		**Control (N = 200)**	**GDM (N = 200)**	**P value** 2
Age (year)^1^		26.6 ± 4.7	29.8 ± 4.6	< 0.001
Familial history of GDM	Positive	18 (9%)	40 (20%)	0.04
	Negative	182 (91%)	160 (80%)	
Previous GDM	Positive	7 (3.5%)	22 (11%)	< 0.001
	Negative	193 (96.5%)	178 (89%)	
Complications during pregnancy	Yes	25 (12%)	12 (6%)	0.2
	No	175 (78%)	188 (94%)	
Labor	Caesarean	70 (35%)	114 (57%)	0.02
	Natural	130 (65%)	86 (43%)	
Gestational age (week)		27.6 ± 1.7	28.3 ± 2.1	0.58
BMI (kg/m^2^)		24.2 ± 4.4	27.4 ± 5.3	< 0.001
FPG (mg/dl)		85 ± 6.4	97.6 ± 5.1	0.001
1hPG (mg/dl)		120.3 ± 15.3	199.8 ± 13.6	< 0.001
2hPG (mg/dl)		106.5 ± 1.8	168.1 ± 2.6	< 0.001
HbA1c (%)		4.1 ± 0.31	5.5 ± 0.66	< 0.001
TC (mg/dl)		207.4 ± 42.6	200.7 ± 46.4	0.24
LDL.C (mg/dl)		124.8 ± 34.9	104.1 ± 34.4	< 0.001
HDL.C (mg/dl)		68.3 ± 19.5	55.7 ± 11.7	0.001
TG (mg/dl)		177.3 ± 27.8	222.8 ± 82.3	< 0.001
Neonate weight (kg)		3.1 ± 0.4	3.3 ± 0.5	0.1
Neonate height (cm)		50 ± 2.2	50.6 ± 2.9	0.2
Neonate head circumference (cm)		35.8 ± 1.7	35.5 ± 1.5	0.4
Apgar		9.03 ± 0.58	9.01 ± 0.69	0.8

1 Means ± SE (all such values);

2 p values were measured by independent sample t-test for quantitative and chi-square test for qualitative data; FPG, fasting plasma glucose; TC, total cholesterol; TG, triglyceride; GDM, gestational diabetes mellitus

## Discussion

According to recent studies, HbA1c value has become globally standardized and been suggested as a simpler, more accurate and automated test for GDM screening (18, 19). However, diagnostic cut-offs reported in different studies are inconsistent (9, 10, 18, 19). 

**Table 2:** HbA1c test performance in GDM diagnosis

**HbA1c**	**Sensitivity (%)**	**Specificity (%)**
4.2500	0.938	0.00
4.3500	0.923	0.031
4.4500	0.908	0.062
4.5500	0.892	0.2
4.6500	0.862	0.323
4.7500	0.846	0.415
4.8500	0.831	0.554
4.9500	0.800	0.692
5.0500	0.800	0.76
5.1500	0.769	0.8
5.2500	0.723	0.877
5.3500	0.662	0.954
5.4500	0.585	0.969
5.5500	0.508	0.969
5.6500	0.431	0.985
5.7500	0.369	1
5.8500	0.292	1
5.9500	0.262	1
6.0500	0.231	1
6.1500	0.169	1
6.2500	0.108	1
6.4000	0.046	1
6.5500	0.031	1

It seems that due to differences among genetic and environmental factors, it is necessary to determine cut-off values for HbA1c in each population. The present study showed that the best cut-off value for HbA1c as a screening tool in Iranian women with GDM is 5.05%. Sensitivity and specificity for HbA1c were 80% and 76%, respectively. The HbA1c cut-off points of 5.3% and 5.4% were reported by WHO 1999 and ADA/WHO 2013 for GDM diagnosis (9, 10). Having said that, sensitivity values for these cut-offs were 68% and 70%, which do not provide robust basis for measure being a standard screening tool. Our results showed that HbA1c values and OGTT in GDM were significantly higher than healthy pregnant women. Age, familial and previous history of GDM, BMI and lipid profile (except total cholesterol) were also significantly different between the two groups. More pregnant women in the GDM group underwent cesarean section. Insulin resistance occurs during pregnancy due to secretion of hormones by the placenta, including growth hormone, corticotropin-releasing hormone, placental lactogen and progesterone. This phenomenon ensures adequate nutrients to the developing fetus (20). GDM occurs when mother has insufficient insulin to cope increasing need. Due to the OGTT disadvantages for GDM screening, HbA1c was suggested as a screening tool and it should be optimized in each population for determine cut-off values. 

In a study done in Indian women, researchers suggested HbA1c ≥ 5.95% as a cut-off value to confirm the diagnosis of GDM (18). The other study compared OGTT and HbA1c for detecting GDM. Researchers concluded that a cut-off value of ≥ 5.4% had 85.7% sensitivity and 61.1% specificity (21). In a smaller retrospective study, accuracy of HbA1c in detecting GDM was 87% compared with OGTT. The cut-off value of HbA1c was 6% in the mentioned study (22). Our study was a case-control study which GDM was diagnosed according to the OGTT. Given that anemia can affect cut-off value of HbA1c, women with anemia was excluded from this study. More patients in the patient group had history of previous GDM. Therefore, it was concluded that the cut-off value of HbA1c in patients with the previous history of GDM is lower than others. Genetic variations lead to differences in HbA1c levels due to variant degree of Hb glycosylation independent of glycaemia (23). Hence, it is proposed that reference ranges should be established before implementation of HbA1c as a universal screening test for GDM. To our knowledge, there is no study that determines cut-off value for HbA1c in Iranian women with known GDM (diasgnosed via OGTT). It is anticipated that validating simple screening tools such as HbA1c in pregnancy lead to decrease in burden of testing and increase in patient’s access and compliance. This would play an important role in the GDM management.

The present study has a few limitations. The most important one was that in this case-control study the causal relationship between variables could not be analyzed. Moreover, participants were from North-west of Iran which may differ from other populations. It is suggested that this study should be done in different ethnic groups in Iran, before the application of HbA1c as a method for GDM screening. In another hand, the present study was done in a referral center of Zanjan. More multi-center studies with larger sample size are needed to consider 5.05% as HbA1c cut-off value for screening of GDM pregnant women in North-west of Iran.

## Conclusion

Pregnant women with HbA1c of ≥ 5.05% should be proceed with an OGTT. But more studies with larger sample size are needed to determine the diagnostic and screening value of HbA1c in identifying pregnant women with GDM.

## References

[1] ADA (2014). American Diabetes Association position statement: gestational diabetes mellitus. Diabetes Care.

[2] Velkoska Nakova V, Krstevska B, Dimitrovski Ch, Simeonova S, Hadzi-Lega M, Serafimoski V (2010). Prevalence of thyroid dysfunction and autoimmunity in pregnant women with gestational diabetes and diabetes type 1. Prilozi.

[3] Buckley BS, Harreiter J, Damm P, Corcoy R, Chico A, Simmons D (2012). Gestational diabetes mellitus in Europe: prevalence, current screening practice and barriers for screening. Diabet Med.

[4] Monk C, Georgieff MK, Osterholm EA (2013). Research review: maternal prenatal distress and poor nutrition—mutually influencing risk factors affecting infant neurocognitive development. J Child Psychol Psychiatry.

[5] Nankervis A, McIntyre HD, Moses RG, P Ross G, K Kallaway L (2013). Testing for gestational diabetes mellitus in Australia. Diabetes Care.

[6] van den Boogaard E, Vissenberg R, Land JA, van Wely M, van der Post JA, Goddijn M (2011). Significance of (sub)clinical thyroid dysfunction and thyroid autoimmunity before conception and in early pregnancy: a systematic review. Hum Reprod Update.

[7] Waugh N, Pearson D, Royle P (2010). Screening for hyperglycaemia in pregnancy: Consensus and controversy. Best Pract Res Clin Endocrinol Metab.

[8] Farrar D, Duley L, Medley N, Lawlor DA (2015). Different strategies for diagnosing gestational diabetes to improve maternal and infant health. Cochrane Database Syst Rev.

[9] American Diabetes Association (2015). (2)Classification and diagnosis of Diabetes. Diabetes Care.

[10] World Health Organization (2011). Use of glycated haemoglobin (HbA1c) in the diagnosis of diabetes mellitus. Abbreviated report of a WHO consultation.

[11] d’Emden M (2014). Glycated haemoglobin for the diagnosis of diabetes. Aust Prescriber.

[12] Selvin E, Crainiceanu CM, Brancati FL, Coresh J (2007). Short-term variability in measures of glycemia and implications for the classification of diabetes. Arch Intern Med.

[13] Agarwal MM, Hughes PF, Punnose J, Ezimokhai M, Thomas L (2001). Gestational diabetes screening of a multiethnic, high-risk population using glycated proteins. Diabetes Res Clin Pract.

[14] Agarwal MM, Dhatt GS, Punnose J, Koster G (2005). Gestational diabetes: a reappraisal of HBA1c as a screening test. Acta Obstet Gynecol Scand.

[15] Pollak A, Brehm R, Havelec L, Lubec G, Malamitsi-Puchner A, Simbrunner G (1981). Total glycosylated hemoglobin in mothers of large-for-gestational-age infants: a postpartum test for undetected maternal diabetes?. Biol Neonate.

[16] Griffiths RJ, Vinall PS, Stickland MH, Wales JK (1987). Haemoglobin A1c levels in normal and diabetic pregnancies. Eur J Obstet Gynecol Reprod Biol.

[17] Moses RG (2012). HbA1c and the diagnosis of gestational diabetes mellitus-a test whose time has not yet come. Diabetes Res Clin Pract.

[18] Breitenbach Renz P, Cavagnolli G, Schwerz Weinert L, Pinho Silveiro S, Lins Camargo J (2015). HbA1c Test as a Tool in the Diagnosis of Gestational Diabetes Mellitus. PLOS ONE.

[19] Khalafallah A, Phuah E, Al-Barazan AM, Nikakis I, Radford A, Clarkson W (2016). Glycosylated haemoglobin for screening and diagnosis of gestational diabetes mellitus. BMJ Open.

[20] Rajput R, Yogesh Yadav, Rajput M, Nanda S (2012). Utility of HbA1c for diagnosis of gestational diabetes mellitus. Diabetes Res Clin Pract.

[21] Aldasouqi SA, Solomon DJ, Bokhari SA, Khan PM, Muneera S, Gossain  VV (2008). Glycohemoglobin A1c: a promising screening tool in gestational diabetes mellitus. Int J Diabetes Dev Ctries.

[22] Butte NF (2000). Carbohydrate and lipid metabolism in pregnancy: normal compared with gestational diabetes mellitus. Am J Clin Nutr.

[23] Lippi G, Targher G (2010). Glycated hemoglobin (HbA1c): old dogmas, a new perspective?. Clin Chem Lab Med.

